# SARS-CoV-2 in pediatric cancer: a systematic review

**DOI:** 10.1007/s00431-021-04338-y

**Published:** 2022-01-10

**Authors:** Sandy Schlage, Thomas Lehrnbecher, Reinhard Berner, Arne Simon, Nicole Toepfner

**Affiliations:** 1 Department of Pediatrics, University Hospital Carl Gustav Carus, Technische Universität, Dresden, Germany; 2 Division of Pediatric Hematology and Oncology, Hospital for Children and Adolescents, Johann Wolfgang Goethe University, Frankfurt, Germany; 3 Pediatric Oncology and Hematology, University Children’s Hospital, Homburg, Saar, Germany

**Keywords:** Pediatric, Cancer, Malignancy, Chemotherapy, SARS-CoV-2, COVID-19

## Abstract

The outbreak of the novel severe acute respiratory syndrome coronavirus 2 (SARS-CoV-2) in December 2019 in Wuhan challenges pediatric oncologists in an unexpected way. We provide a comprehensive overview, which systematically summarizes and grades evidence (QoE) on SARS-CoV-2 infections in pediatric cancer patients at 1.5 years of pandemic. A systematic literature search in PubMed combined with an additional exploratory literature review in other international databases was conducted to identify studies on children (aged < 18 years) with a malignant disease and COVID-19 infections. In total, 45 reports on 1003 pediatric cancer patients with SARS-CoV-2 infections were identified out of 1397 reports analyzed. The clinical course of COVID-19 was reported mild or moderate in 358 patients (41.7%), whereas 11.1% of patients showed severe COVID-19. In 12.7% of patients, chemotherapy was postponed, whereas 19% of patients with different underlying malignancies received chemotherapy during SARS-CoV-2 infection. Twenty-five patients with SARS-CoV-2 infections died, potentially related to COVID-19.

*Conclusion*: Despite a favorable COVID-19 outcome in most pediatric cancer patients, the morbidity is reported higher than in children without comorbidities. However, no severe COVID-19 complications were associated to the continuation of chemotherapy in some cohort studies and reports on two patients. Therefore, the risk of cancer progress or relapse due to interruption of chemotherapy has carefully to be weighed against the risk of severe COVID-19 disease with potentially fatal outcome.

**Table:** 

**What is Known:** *• Most of pediatric patients with malignant diseases show an asymptomatic, mild or moderate clinical course of SARS-CoV-2 infection. * *• Current need for a basis for decision-making, whether to stop or interrupt cancer treatment in a patient infected with SARS-CoV-2, and when to continue chemotherapy.*	
**What is New:** *• Review results comprising over 1000 pediatric COVID-19 cancer patients confirm mild courses of SARS-CoV-2 infection in most patients but also show the attributable mortality is at least 10 times higher compared to reports on hospitalized children without comorbidities.* *• Review identifies that chemotherapy was continued despite SARS-CoV-2 positivity in 18% of patients with individual chemotherapy modification according to the clinical course of SARS-CoV-2 infection and existing comorbidities. On this basis, no severe COVID-19 complications were associated to the continuation of chemotherapy in several cohort studies and two case reports.*

**Supplementary information:**

The online version contains supplementary material available at 10.1007/s00431-021-04338-y.

## Introduction

Pandemic caused by severe acute respiratory syndrome coronavirus-2 (SARS-CoV-2) has considerably affected pediatric oncology services worldwide. Multiple SARS-CoV-2-related effects on both population and individual patient levels have emerged. For example, delayed hospital admissions or the reduced availability of chemotherapeutic drugs have led to substantial disruptions of cancer diagnosis and management [1–3]. In addition, it remained unclear, which cancer patients were at high risk for a severe clinical course, whether to stop or interrupt cancer treatment in a patient infected with SARS-CoV-2, and when to continue therapy, all questions which might have an important impact on overall outcome.

In the early pandemic phase, children with malignancies undergoing cancer-directed therapy were presumed to be at higher risk for severe COVID-19 due to their immunocompromised state. In the meantime, many case reports and cohort studies from national and international registries confirmed that the majority of pediatric cancer patients experience only clinically mild to moderate symptoms related to their SARS-CoV-2 infection [4–20], although it is clear that severe COVID-19 events and even fatalities may occur.

The aim of this systematic review was to update the available information on SARS-CoV-2 infections in pediatric cancer patients and to grade existing evidence as defined by the European Society of Clinical Microbiology and Infectious Diseases (ECCMID) [28] to optimize the rational management for this vulnerable group of patients.

## Materials and methods

### Systematic search strategy

A systematic search was conducted in PubMed on October 7, 2021, with search terms combined by Boolean operators (AND, OR) and truncated search terms according to the PubMed User Guide. PubMed’s Automatic Term Mapping was applied and the following MESH terms were used: ((newborn) OR (neonat*)) OR (infant*) OR (toddler*) OR (pre-schooler) OR (preschooler) OR (child*) OR (children) OR (adolescen*) OR (pediatr*) OR (paediatr*) OR (youth*) OR (teen*) OR (kid*) OR (bab*) AND (((coronavirus*)) OR (corona virus)) OR (COVID-19*) OR (COVID 19) OR (SARS-Cov-2) OR (SARS Cov 2) OR (MERS*) OR (SARS* pandemic*) AND ((immunosuppre*) OR (immunocomp*)) AND ((tumor*) OR (tumour) OR (solid tumor) OR (solid tumour) OR (cancer) OR (leukaemia) OR (leukemia) OR (transplantation) OR (HSCT) OR (chemother*)).

All manuscripts published after the onset of SARS-CoV-2 pandemic (December 2019) were included. Search results were narrowed by the following filters (primary review inclusion criteria): species = human and language = english. Studies were included if patients were pediatric patients as defined by age up to 18 years. References were included in the analysis if SARS-CoV-2-positive patients had an underlying malignant disease and had received or were receiving immunosuppressive therapy. Detections of SARS-CoV-2 by rtPCR or Rapid Antigen Detection Test (RADT) were accepted. Two reviewers (SaS and NT) independently evaluated the titles and abstracts of publications identified by the search strategy, and all potentially relevant publications were retrieved in full text. The final decision to include studies into the systematic review was consented by all authors. According to the Guideline by the Infectious Diseases Working Party (AGIHO) of the German Society for Haematology and Medical Oncology (DGHO) [27], we added the quality of evidence (QoE) following the grading system proposed by the European Society of Clinical Microbiology and Infectious Diseases (ECCMID) [28]. We additionally added codes for QoE level III to provide most comprehensive grading information. The codes for QoE level III were defined as follows: RA opinions of respected authorities, EC consensus of expert committees, CE based on clinical experiences of experts, CS based on descriptive case studies and CR based on case reports.

### Exploratory literature searches

An additional exploratory literature search was conducted during the period December 2019 to October 2021 with the following keywords: pediatrics, paediatrics, child, children, infant, toddler, scholar, adolescent, COVID-19, COVID, SARS-CoV-2, SARS, coronavirus, corona pandemic, cancer, leukaemia, leukemia, tumor, malignancy, malignant, and immunosuppression. The following information sources were used: MEDLINE, Embase, Orphanet, Wiley Online Library, The Cochrane Library, Google Scholar, Authoria, Oxford Academic, TRIP Database, MedRxiv, and BioRxiv. Further literature search was conducted in the following journals: European Journal of Pediatrics, JAMA, Journal of Pediatric Infectious Diseases, Nature, New England Journal of Medicine, Pediatric Blood & Cancer, Science, and The Lancet. Additionally, relevant studies were identified from the reference lists of all retrieved full text articles.

### Procedure of data analysis

After matching the review search inclusion criteria, the eligibility of all retrieved studies and reports was assessed on full text manuscripts. During this process, 45 publications were evaluated via modified consort criteria for transparent reporting. From all manuscripts meeting the eligibility criteria, data were obtained and integrated into evidence tables (Table 1 and Online Resource 1). The clinical courses of SARS-CoV-2 infections of all pediatric cancer patients were classified according to Dong et al. [32].

**Table 1:** Fifteen studies on pediatric COVID cancer patients

***Title*** ***Doi*** ***Date of online publication***	***Study design*** ***Quality of evidence (QoE)***	***Number of patients with SARS-CoV-2 infection: all patients (n***_***all***_***)*** ***Cancer patients (n***_***cc***_***)*** ***Gender (out of (n***_***cc***_***)*** ***Median age***	***Continent*** ***of origin*** ***Ethnicity***	***Type of malignancy***	***Presented course of SARS-CoV-2 infection***	***Modification on cancer treatment***	***Treatment of SARS-CoV-2***	***Outcome***	***Conclusions***
*SARS-CoV-2 in children with cancer or following hematopoietic stem cell* *10.1016/j.ejca.2021.09.027* *09.10.2021*	Multicentric study II_T_	*n*_all_ = 131 *n*_cc_ = 131 Male *n* = 47 8 years	Europe Caucasian	Leukemia *n* = 60 Lymphoma *n* = 18 Solid tumor *n* = 48 Post-HSCT *n* = 5	Asymptomatic *n* = 42 Mild *n* = 61 Moderate *n* = 11 Severe/critical *n* = 17 Not reported *n* = 0	Postponed/delay *n* = 30 Continued *n* = 63 Modified *n* = 6 Not reported *n* = 27	Antiviral treatment *n* = 11	Recovered *n* = 2 Death *n* = 4 Not reported *n* = 126	Chemotherapy modification according to the clinical course of SARS-CoV-2 infection and existing comorbidities
*SARS-CoV-2 persistence in immunocompromised children* *10.1002/pbc.29277* *28.08.2021*	Cohort study II_U_	*n*_all_ = 91 *n*_cc_ = 48 Male *n* = 58 15.5 years	North America Hispanic or Latinx Caucasian	Solid tumor *n* = 10 Leukemia Lymphoma *n* = 31 Post-HSCT *n* = 4 Others *n* = 13	Severe/critical *n* = 3 Not reported *n* = 45	No further information	No further information	Death *n* = 4 Not reported *n* = 47	Routine PCR-based SARS-CoV-2-screening in immunocompromised children to guide the management and the ongoing risk of transmission
*Management and outcome of coronavirus disease 2019 (COVID-19) in pediatric cancer patients: a single-center experience from a developing country* *https://doi.org/10.1016/j.clml.2021.07.025* *26.07.2021*	Cohort study I_U_	*n*_all_ = 76 *n*_cc_ = 76 Male *n* = 42 9 years	Africa African	ALL/LL *n* = 38 AML *n* = 20 Lymphoma *n* = 5 CML *n* = 3 Neuroblastoma *n* = 3 RMS/NRMS *n* = 3	Asymptomatic *n* = 0 Mild *n* = 6 Moderate *n* = 43 Severe/critical *n* = 27 Not reported *n* = 0	Postponed/delay *n* = 26 Continued *n* = 0 Modified *n* = 23 Not reported *n* = 27	Remdesivir *n* = 45 No remdesivir *n* = 31	Recovered *n* = 66 Death *n* = 10 Not reported *n* = 0	Modification of chemotherapy according to the course of infection Antiviral treatment could be beneficial in managing severe courses of SARS-CoV-2 infection
*Initial report on Spanish pediatric oncologic, hematologic, and post-stem cell transplantation patients during SARS-CoV-2 pandemic* *10.1002/pbc.28557* *16.07.2020*	Cohort study II_T_	*n*_all_ = 47 *n*_cc_ = 47 Male *n* = 34 8.2 years	Europe Caucasian	Nonmalignant hemopathy *n* = 6 Solid tumor *n* = 14 Leukemia/lymphoma *n* = 19 Post-HSCT *n* = 8	Asymptomatic *n* = 12 Mild *n* = 24 Moderate *n* = 0 Severe/critical *n* = 11 Not reported *n* = 0	Postponed/delay *n* = 22 Continued *n* = 24 Modified *n* = 0 Not reported *n* = 1	Hydroxychloroquine *n* = 23	Recovered *n* = 46 Death *n* = 1 Not reported *n* = 0	Multidisciplinary discussions for decisions on anticancer treatment delay
*Clinical characteristics and outcomes of a cohort of pediatric oncohematologic patients with COVID-19 infection in the City of Bogotá, Colombia* *10.1097/INF.0000000000003135* *01.06.2021*	Cohort study II_U_	*n*_all_ = 33 *n*_cc_ = 33 Male *n* = 21 10 years	Europe Caucasian	ALL *n* = 16 Medulloblastoma *n* = 3 AML *n* = 3 Lymphoma *n* = 4 Pinealoblastoma *n* = 1 Osteosarcoma *n* = 1 Ewing’s sarcoma *n* = 1 Wilms’ tumor *n* = 1 Germ cell tumor *n* = 1 Sacrococcygeal teratoma *n* = 1 Bone marrow failure *n* = 1	Asymptomatic *n* = 8 Mild *n* = 7 Moderate *n* = 0 Severe/critical *n* = 7 Not reported *n* = 11	Postponed/delay *n* = 6 Continued *n* = 0 Modified *n* = 0 Not reported *n* = 27	No further information	Recovered *n* = 0 Death *n* = 2 Not reported *n* = 31	Establishment of isolation protocols for SARS-CoV-2-positive patients Multidisciplinary decision on cancer treatment continuation, modification, or postpone or SARS-CoV-2-positive patients
*Clinical characteristics and outcome of severe acute respiratory syndrome coronavirus 2 infection in Italian pediatric oncology patients: a study from the Infectious Diseases Working Group of the Associazione Italiana di Oncologia e Ematologia Pediatrica* *10.1093/jpids/piaa088* *10.11.2020*	Cohort study II_U_	*n*_all_ = 29 *n*_cc_ = 29 Male *n* = 13 7 years	Europe Caucasian	Leukemia *n* = 16 Lymphoma *n* = 3 Ewing sarcoma *n* = 1 Hepatoblastoma *n* = 2 Wilms tumor *n* = 1 CNS tumor *n* = 1 RMS *n* = 1 Other *n* = 2	Asymptomatic *n* = 18 Mild *n* = 7 Moderate *n* = 4 Severe/critical *n* = 0 Not reported *n* = 0	Postponed/delay *n* = 16 Continued *n* = 8 Modified *n* = 2 Not reported *n* = 3	Hydroxychloroquine *n* = 9 Lopinavir/ritonavir *n* = 3	Recovered *n* = 29 Death *n* = 0 Not reported *n* = 0	Avoidance of major changes to planned anticancer treatment
*COVID-19 in children with cancer in low- and middle-income countries: experience from a cancer center in Chennai, India* *10.1080/08880018.2020.1831113* *05.11.2020*	Case series III_CS_	*n*_all_ = 15 *n*_cc_ = 15 Male *n* = 9 9.4 years	Asia Asian	ALL *n* = 8 AML *n* = 2 Hepatoblastoma *n* = 2 MPAL *n* = 2 Wilms’ tumor *n* = 1	Asymptomatic *n* = 8 Mild *n* = 0 Moderate *n* = 6 Severe/critical *n* = 1 Not reported *n* = 0	Postponed/delay *n* = 0 Continued *n* = 0 Modified *n* = 0 Not reported *n* = 15	No further information	Recovered *n* = 15 Death *n* = 0 Not reported *n* = 0	Routine testing of cancer patients and caregivers for SARS-CoV-2
*COVID-19 infection in pediatric recipients of allogeneic stem cell transplantation: the UK experience* *10.1111/bjh.17547* *20.06.2021*	Case series III_CS_	*n*_all_ = 9 *n*_cc_ = 5 Male *n* = 3 12 years	Europe Caucasian/Asian/African	ALL *n* = 2 AML *n* = 2 Lymphoma *n* = 1 Other *n* = 4	Asymptomatic *n* = 2 Mild *n* = 6 Moderate *n* = 1 Severe/critical *n* = 0 Not reported *n* = 0	Postponed/delay *n* = 0 Continued *n* = 0 Modified *n* = 0 Not reported *n* = 9	No further information	Recovered *n* = 8 Death *n* = 1 Unrelated to SARS-CoV-2 Not reported *n* = 0	Screening for SARS-CoV-2 despite identification of other pathogens
*Flash survey on severe acute respiratory syndrome coronavirus-2 infections in pediatric patients on anticancer treatment* *10.1016/j.ejca.2020.03.021* *07.04.2020*	Case series III_CS_	*n*_all_ = 9 *n*_cc_ = 8 Male *n* = 3 3.5 years	Europe Caucasian	ALL *n* = 2 Osteosarcoma *n* = 1 Hepatoblastoma *n* = 1 Cervical rhabdoid tumor *n* = 1 Ewing sarcoma *n* = 1 Wilms’ tumor *n* = 1 Solid tumor *n* = 1	Asymptomatic *n* = 0 Mild *n* = 6 Moderate *n* = 2 Severe/critical *n* = 0 Not reported *n* = 0	Postponed/delay *n* = 0 Continued *n* = 0 Modified *n* = 0 Not reported *n* = 8	Hydroxychloroquine *n* = 2 Lopinavir/ritonavir *n* = 1	Recovered *n* = 5 Death *n* = 0 Not reported *n* = 3	Preventive measures against SARS-CoV-2 pandemics should not cause delays in oncological treatment despite they are essential to avoid transmissions
*High mortality of COVID-19 in children with cancer in a single center in Algiers, Algeria* *10.1002/pbc.28898* *19.02.2021*	Case series III_CS_	*n*_all_ = 7 *n*_cc_ = 7 Male *n* = 3 5 years	Africa African	Leukemia *n* = 5 Lymphoma *n* = 1 Neuroblastoma *n* = 1	Asymptomatic *n* = 3 Mild *n* = 0 Moderate *n* = 4 Severe/critical *n* = 0 Not reported *n* = 0	Postponed/delay *n* = 7 Continued *n* = 0 Modified *n* = 0 Not reported *n* = 0	Hydroxychloroquine *n* = 3	Recovered *n* = 5 Death *n* = 0 Not reported *n* = 3	Especially in limited resource settings cancer patients are a potentially vulnerable group for worse outcomes Differences in the use of critical care resources might influence the outcome of cancer patients with SARS-CoV-2 infections Early identification of severe SARS-CoV-2 infection courses and early supportive medical care is important
*Benign course of SARS-CoV-2 infection in a series of pediatric oncology patients* *10.1002/pbc.28504* *23.06.2020*	Case series III_CS_	*n*_all_ = 6 *n*_cc_ = 6 Male *n* = 2 8 years	North America Caucasian	ALL *n* = 2 AML *n* = 1 Osteosarcoma *n* = 1 Mixed germ cell tumor *n* = 1 Lymphoma *n* = 1	Asymptomatic *n* = 2 Mild *n* = 2 Moderate *n* = 2 Severe/critical *n* = 0 Not reported *n* = 0	Postponed/delay *n* = 0 Continued *n* = 0 Modified *n* = 0 Not reported *n* = 6	No further information	Recovered *n* = 6 Death *n* = 0 Not reported *n* = 0	Careful administration of anticancer therapy in patients with a mild course of SARS-CoV-2 infection is an option
*Remdesivir during induction chemotherapy for newly diagnosed pediatric acute lymphoblastic leukemia with concomitant SARS-CoV-2 infection* *10.1111/bjh.17014* *17.08.2020*	Case report III_CR_	*n*_all_ = 1 *n*_cc_ = 1 Male *n* = 1 5 years	Europe Caucasian	ALL *n* = 1	Asymptomatic *n* = 2 Mild *n* = 0 Moderate *n* = 0 Severe/critical *n* = 1 Not reported *n* = 0	Postponed/delay *n* = 0 Continued *n* = 1 Modified *n* = 0 Not reported *n* = 0	Remdesivir *n* = 1	Recovered *n* = 1 Death *n* = 0 Not reported *n* = 0	Antiviral therapy with remdesivir may be helpful to shorten the time of recovery and fasten the begin of anticancer treatment
*Screening of SARS-CoV-2 in 299 hospitalized children with hemato-oncological diseases: a multicenter survey in Hubei, China* *10.1007/s11596-020–2228-7* *01.05.2020*	Cross-sectional study II_U_	*n*_all_ = 299 *n*_cc_ = 1 Male *n* = 1 8 years	Asia Asian	ALL *n* = 1	Asymptomatic *n* = 2 Mild *n* = 0 Moderate *n* = 0 Severe/critical *n* = 1 Not reported *n* = 0	Postponed/delay *n* = 0 Continued *n* = 0 Modified *n* = 0 Not reported *n* = 1	No further information about the one confirmed SARS-CoV-2 case	Recovered *n* = 1 Death *n* = 0 Not reported *n* = 0	Strict adherence to effective hygiene measures is important (hand hygiene, social distance in public places, wearing masks correctly) Protective measures against SARS-CoV-2 and ward management for risk reduction
*A 10-year-old girl with late acute lymphoblastic leukemia recurrence diagnosed with COVID-19 and treated with remdesivir* *10.1097/MPH.0000000000002166* *21.04.2021*	Case report III_CR_	*n*_all_ = 1 *n*_cc_ = 1 Male *n* = 0 10 years	Europe Caucasian	ALL *n* = 1	Asymptomatic *n* = 0 Mild *n* = 0 Moderate *n* = 1 Severe/critical *n* = 0 Not reported *n* = 1	Postponed/delay *n* = 1 Continued *n* = 0 Modified *n* = 0 Not reported *n* = 0	Remdesivir *n* = 1	Recovered *n* = 1 Death *n* = 0 Not reported *n* = 0	More research needed to find optimal treatment regimens especially for children with high-risk factors for severe courses of SARS-CoV-2 infections Until effective vaccines are available preventive actions reducing the risk of infections are of highest priority
*Severe COVID-19 infection in a child receiving immunotherapy for cancer* *10.1002/pbc.28710* *01.03.2021*	Case report III_CR_	*n*_all_ = 1 *n*_cc_ = 1 Male *n* = 0 23 months	North America Caucasian	Neuroblastoma *n* = 1	Asymptomatic *n* = 0 Mild *n* = 0 Moderate *n* = 0 Severe/critical *n* = 1 Not reported *n* = 0	Postponed/delay *n* = 1 Continued *n* = 0 Modified *n* = 0 Not reported *n* = 0	Remdesivir *n* = 1	Not reported *n* = 0	Modification of anticancer treatment according to the severity of SARS-CoV-2 infection course

*ALL* acute lymphoblastic leukemia, *AML* acute myeloid leukemia, *CML* chronic myeloid leukemia, *MPAL* mixed phenotypic acute leukemia, *NRMS* non-rhabdomyosarcoma, *RMS* rhabdomyosarcoma

## Results

### Cohort analysis of SARS-CoV-2-infected patients

**Fig. 1:**
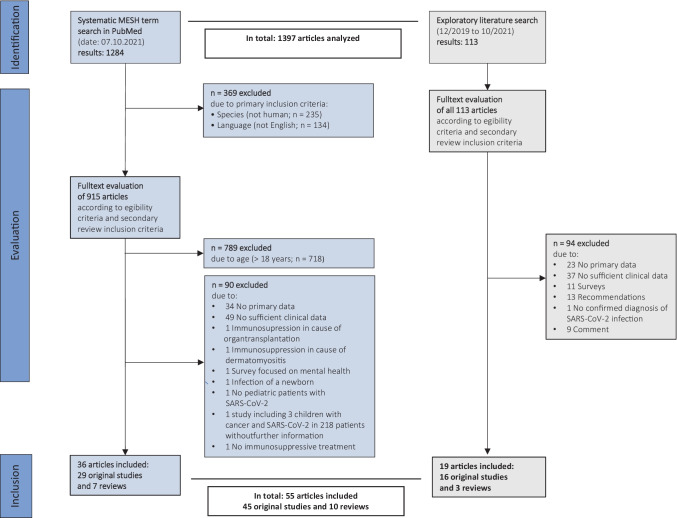
Results of the systematic and non-systematic searches and the decisions concerning the inclusion and exclusion of the retrieved articles

The results of the systematic and non-systematic searches and the decisions concerning the inclusion and exclusion of the retrieved articles are described in Fig. 1. A total of 45 original articles and ten reviews were retrieved from 1397 articles analyzed. The original studies include 1 survey, 10 case series, 19 case reports, 3 prospective cohort studies, 7 retrospective cohort studies, 4 multicentric studies, and 1 cross-sectional cohort study. The dates of online publications ranged between April 2020 and October 2021.

Data from pediatric cancer patients of 5 continents (Europe, North America, South America, Asia, Africa) were assessed comprising 39 cohorts of children below 18 years of age and 6 cohorts of children and adults. In these studies, the number of all SARS-CoV-2 patients reported varied between a case report on one patient and 179 patients described in a multicentric cohort study (QoE between III_CR_ and II_T_, median: 24 patients per study). The reason for SARS-CoV-2 testing was only sporadically reported. In 33 studies, SARS-CoV-2 infections were identified by nasopharyngeal swabs (73.3%), in one study by throat swabs (4.4%), and ten studies did not comment on the material for SARS-CoV-2-detection (22.2%). SARS-CoV-2-positive results were obtained by rtPCR in 41 studies (91.1%). In all studies no SARS-CoV-2 variants were reported. Twenty one of 45 publications reported on inpatients with SARS-CoV-2 infection (46.7%) as well as one publication reported on outpatients (2.2%). In 23 studies, patients received inpatient and outpatient medical care during SARS-CoV-2 infection (51.1%). An overview on the study results of 15 studies on pediatric COVID cancer patients is presented in Table 1.

### Analysis of individual SARS-CoV-2 patients

In total, 79 patients with SARS-CoV-2 infection were retrieved from all reviewed studies, including studies with QoE II_U_, II_T_, II_CR_, and III_CS_. Of these 79 patients, 45 were male (57.1%), 17 female (33.0%), and the gender of 8 patients (10.1%) was not reported. The median age was 8 years and varied between 6 months and 17 years.

### Types of underlying malignancy

The most common malignant disease reported was acute lymphoblastic leukemia (ALL) in 40 patients (50.1%), followed by AML in eight patients (10.1%). Four patients each (5.1%) had malignant teratoid rhabdoid tumor, Wilms’ tumor, hepatoblastoma or lymphoma. Three patients each (3.8%) had osteosarcoma or neuroblastoma. Two patients were reported with melanoma (2.5%) and two patients with myelodysplastic syndrome (2.5%). One patient had a CNS glioma (1.3%), and one patient had a mixed germ cell tumor (1.3%). Four patients acquired the SARS-CoV-2 infection 3 to 5 months after hematopoietic stem cell transplantation (5.1%). Three of these patients suffered from graft-versus-host disease (one male and two female patients, all with grade III) [21–23].

### Data on chemotherapy

During the time of SARS-CoV-2 infection onset, 55 patients received first-line intensive chemotherapy or were on oral maintenance therapy (69.6%), 15 patients were in remission (18.9%), 8 patients had progressive disease and received various individual rescue treatment regimens (10.8%), and one patient had refractory malignant diseases and was on palliative oral chemotherapy (1.5%).

### Origin of the SARS-CoV-2 infection

In 80.0% of all patients (*n* = 63) the origin of SARS-CoV-2 infection was not reported, whereas in 16 patients (20.3%) the infection was thought to be acquired from family members [6–8, 11, 12, 21, 22, 29–32]. In 4 patients (5.1%) a possible reinfection was documented [5, 9, 11].

### Clinical symptoms and morbidity of COVID-19

At diagnosis of the SARS-CoV-2 infection, 22 patients were asymptomatic (27.8%), 56 patients (70.9%) presented with the following symptoms: fever (*n* = 49; 59.5%), dyspnea (*n* = 20; 25.3%), cough (*n* = 17; 21.5%), sore throat (*n* = 7; 8.7%) and gastrointestinal symptoms such as diarrhea (*n* = 5; 6.3%). Out of all 71 patients with information available, the clinical course was mild in 44 patients (62.0%), moderate in 13 patients (18.3%), and severe in 14 patients (19.7%). Five patients (7.0%) died during the period of observation, one with ALL (no further details) [33], one with osteosarcoma and extensive pulmonary metastases [34], 2 with COVID-19-related multiorgan failure after SCT and CAR-T-cell therapy [23], and one patient with neuroblastoma [35]. Additionally, 15 deaths of SARS-CoV-2-positive pediatric cancer patients were reported without mentioning the underlying malignancy [38–41] and 5 deaths related to SARS-CoV-2 associated complications additive to refractory ALL [36, 37] were reported.

### Peripheral blood counts and laboratory parameters

The total leucocyte counts were mentioned in 19 patients (24.1%) ranging from 0.8 to 9.6 × 10^9^/l (median: 1.2 × 10^9^/l). The absolute neutrophil counts were reported in 15 patients (19.0%) ranging from 0.1 to 7.4 × 10^9^/l (median: 0.8 × 10^9^/l). Additionally, 37 patients were reported to be neutropenic without more details (46.8%). The absolute lymphocyte counts were mentioned in 16 patients (21.7%) ranging from 0.08 to 2.7 × 10^9^/l (median: 1.2 × 10^9^/l). The C-reactive protein values (displayed in 14 patients; 17.2%) ranged between 0.6 and 500.4 mg/l (median: 68.2 mg/l). Interleukin-6 (IL-6) values (6 patients; 7.6%) ranged from 0.86 to 41 pg/ml (median: 9.6 pg/ml). Ferritin values (7 patients; 8.7%) ranged from 622 to 6417 ng/ml (median: 2092 ng/ml). D-dimer levels (9 patients; 11.4%) ranged from 20.6 to 3352 ng/ml (median: 800 ng/ml). Serum SARS-CoV-2 specific antibodies were detected positive in ten patients (12.7%), negative in four patients (5.1%), and not reported in 65 patients (82.3%).

### Duration of COVID-19 symptoms and time to negative PCR

COVID-19 related symptoms lasted less than 7 days in 11 patients (13.9%), in 22 patients between 7 and 14 days (27.8%). In 33 patients, the duration of clinical symptoms was not reported (41.8%). SARS-CoV-2 positivity (by rtPCR; reported in 21 patients) lasted up to 7 days in three patients (3.8%), between 7 and 14 days in 9 patients (11.4%), 15 to 30 days in 3 patients (3.8%), and more than 30 days in 4 patients (5.1%).

### Treatment of COVID-19

Six patients were only in quarantine at home without specific treatment (7.6%), 13 patients (16.5%) were admitted to hospital for observation and 12 patients were transferred to the intensive care unit (15.2%). Twenty-one patients were treated symptomatically with antipyretics (26.6%), ten patients received oxygen (12.7%), five systemic anticoagulation (6.3%), seven patients received antibiotic treatment (8.9%), five patients received corticosteroids (6.3%) and three neutropenic patients received granulocyte-colony-stimulating factor (3.8%). Twenty-five patients received hydroxychloroquine (31.6%) and 16 patients received antivirals (20.3%; five patients lopinavir [6.3%], eight patients received remdesivir [10.1%], one patient acyclovir [1.3%], one patient valganciclovir [1.3%], and one patient oseltamir [1.3%]).

### Modification of the chemotherapy regimen

In 28 patients out of the 79 patients reported in detail (35.4%) information on modification of chemotherapy during the SARS-CoV-2 infection was available. Chemotherapy was interrupted for up to 7 days in 14 patients (17.7%), until a SARS-CoV-2 negative rtPCR result was obtained. In 14 patients (17.7%), chemotherapy was continued regularly. Of the latter, 1 patient received adjuvant chemotherapy for hepatoblastoma (1.3%) [20], 1 patient underwent chemotherapy for cervical rhabdoid tumor (1.3%) [53], and 1 patient with ALL received dexamethasone, vincristine, PEG asparaginase, and intrathecal MTX (1.3%) [42], respectively. One patient received vincristine-daunorubicin (1.3%) [16], and 1 patient received the third cycle of high-risk AML therapy (1.3%) [6]. One patient received oral chemotherapy following the AALL1131 protocol guideline (1.3%) [8]. One ALL patient in remission received dasatinib in reduced dosage (1.3%) [10]. One patient received daily cyclosporine and prednisolone after HSCT (1.3%) [21]. One patient with standard risk ALL received maintenance 6-mercaptopurine and MTX (1.3%) [14]. One AML patient received rituximab before HSCT (1.3%), and two patients received cytarabine during treatment of Hodgkin lymphoma (2.5%).

In 2 patients without therapy modification, no SARS-CoV-2 related complications were observed. In the other 14 patients the detailed clinical courses were not described. In 51 patients (64.6%), no data concerning treatment modification were available. The clinical courses of both SARS-CoV-2-positive patients without chemotherapy modification were as follows: in a 17-year-old girl the SARS-CoV-2 PCR was positive at day 0 after HSCT in cause of AML subtype 5, treated by Mye child high-risk protocol [21]. She only had mild rhinitis and showed no other viral reactivation. She developed a GVHD grade III (cutaneous and digestive) treated with corticosteroids. After 3 months her bone marrow showed a complete donor chimerism. After the positive SARS-CoV-2 PCR result, her treatment with prednisolone (0.4 mg/kg/day), cyclosporine (4 mg/kg/day), and ACE inhibitors (0.12 mg/kg/day) was not postponed. She received intravenous immunoglobulins and her preventive antiinfectious treatment was regularly continued with sulfamethoxazole-trimethoprime, posaconazole, phenoxymethylpenicillin, and valacyclovir. The chest CT revealed scattered ground-glass opacities on day 7. She remained SARS-CoV-2-PCR-positive on days 21 and 42. Anti-SARS-CoV-2-IgM antibodies could be detected on day 14 and remained positive on day 56. Anti-SARS-CoV-2-IgG antibodies were positive on day 56. All in all, it was reported as a mild course of infection. The other patient was a 5-year-old boy with precursor B cell ALL with standard risk [42]. He presented with fever and petechiae. Clinical neck swelling associated with swollen lip and tongue as well as with inspiratory stridor while oxygen saturation was normal. His chest ray showed peribronchial sickening. His TWCC was 6.76 × 10^9^/l. He was treated with remdesivir for 5 days in parallel to the start of ALL induction therapy. He was monitored by daily blood tests. On day 3 an increased ALT was recognized, which peaked at 408 U/l on day 5. In summary, a mild course was reported, and he could be discharged home on day 8. At the end of induction therapy, bone marrow showed a morphological remission with undetectable minimal residual disease. SARS-CoV-2-PCR also remained negative.

## Discussion

To the best of our knowledge, this review comprises the largest cohort reported on pediatric cancer patients with COVID-19 summarizing evidence of 45 articles after systematic literature search and comprehensive analysis of in total 1397 articles at the time of 1.5 years of SARS-CoV-2 pandemic. In comparison to previous reviews, this review focuses unambiguously on pediatric cancer patients and provides quality of evidence levels (QoE) assigned to every study included in the review. The QoE definitions were used as proposed by the European Society of Clinical Microbiology and Infectious Diseases [28] and supported for adult cancer patients by the Guideline by the Infectious Diseases Working Party (AGIHO) of the German Society for Haematology and Medical Oncology (DGHO) [27]. With respect for this grading system and the intention for its best possible use, we added extra codes for QoE level III, which we also suggest using in future for providing most comprehensive grading information for recommendations.

Our meta-analysis revealed that out of 1003 reported pediatric cancer patients with SARS-CoV-2 infection, 23.9% of patients were asymptomatic and the clinical courses of COVID-19 were mild or moderate in 41.7%. In 11.1% of patients the clinical courses of COVID-19 were severe, and 25 patients (2.5%) eventually died related to COVID-19. These results point towards a more favorable situation in pediatric cancer patients compared to adults with malignant disease (QoE II_T_) [43], most probably due to a lesser prevalence of other underling conditions and comorbidities, and due to the higher risk of an adverse outcome of SARS-CoV-2 infection in patients over 65 years (QoE II_T_) [44]. However, these data do not rely on prospective cohort studies in which all pediatric cancer patients are regularly screened for SARS-CoV-2. Therefore, there is no tentative denominator to comment on COVID-19 related hospitalization rates, morbidity and mortality (QoE III_EC_) [48], and the true incidence of SARS-CoV-2 detection and COVID-19 in pediatric cancer patients remains unknown. Of note, despite a mild course of SARS-CoV-2 infection was reported in most of the cancer patients (QoE II_T_ [40], QoE II_T_ [41], QoE II_T_ [46], QoE II_T_ [47]), the attributable mortality of 6.7% is at least 10 times higher compared to reports on hospitalized children without comorbidities (QoE II_T_) [44]. However, most studies (QoE III_CR_ [10], QoE II_T_ [23], QoE III_CR_ [34], QoE III_CR_ [35], QoE III_CR_ [18], QoE II_U_ [38], QoE II_U_ [39], QoE II_T_ [40], QoE II_U_ [49]) did not clearly differentiate between deaths related to COVID-19 and death due to cancer progression. Only two studies explicitly report on one patient out of 54 as well as on four patients out of five with COVID-19-related deaths, respectively (QoE II_U_ [48], QoE II_T_ [41]). One multicenter cohort study reported on an increased mortality of pediatric patients who had completed cancer treatment or had undergone HSCT compared with patients on active treatment (QoE II_T_) [41]. An Algerian study reported on a very high case fatality rate of 28% and pointed out that differences in the use of critical care resources could have influenced the outcome of cancer patients with SARS-CoV-2 infections, which potentially increases the vulnerability of cancer patients in limited resource settings (QoE III_CS_) [36]. In many of the reviewed studies, the reason for SARS-CoV-2 testing was not mentioned. One large cohort study (QoE II_U_) [50] and one case series (QoE III_CS_) [20] recommend routine PCR-based SARS-CoV-2 screening in immunocompromised children to guide management and the ongoing risk of transmissions. Especially when the incidence of newly diagnosed SARS-CoV-2 infections is high in the attending region, an admission screening is performed in most pediatric cancer centers (QoE II_T_) [51]. This approach seems reasonable and may explain the detection of SARS-CoV-2 in asymptomatic patients. In line, our meta-analysis on individual patients revealed 27.8% asymptomatic SARS-CoV-2-positive cancer patients in comparison to 70.9% cancer patients showing clinical symptoms at the time of SARS-CoV-2 positivity (QoE II_U_, II_T_, III_CS_, II_CR_). If clinical symptoms (such as fever or gastrointestinal symptoms) are attributes of SARS-CoV-2 infection or common side effects in pediatric cancer patients receiving chemotherapy may be difficult to differentiate.

Regarding SARS-CoV-2 infection route, it was reported that most SARS-CoV-2 infections in children derive from a close adult contact (e.g., family or household member; QoE III_CR_ [5], QoE III_CR_ [6], QoE III_CS_ [20], QoE III_CR_ [21], QoE II_U_ [30]). In the reviewed dataset, 16 patients (20.3%) acquired the infection from family members, whereas in 86.4% of all patients the origin of SARS-CoV-2 infection was not reported. However, these data underline the importance to provide the whole family with detailed information on preventive strategies (QoE II_U_) and the need for SARS-CoV-2 vaccination of all close contact adults (including all healthcare workers) and adolescents (QoE II_T_). Many studies recommended strict adherence to effective hygiene measures (such as hand hygiene, social distance in public places, wearing masks correctly) and ward management for risk reduction of SARS-CoV-2 transmission (QoE II_U_ [30, 50]).

Besides the complex issues of infection prevention in pediatric oncology units, several reports on different treatment options against COVID-19 exist in pediatric cancer patients [24–26, 36, 45, 49, 53]. Whereas early studies reported on hydroxychloroquine administration (QoE III_CS_ [36], QoE II_U_ [49], QoE III_CS_ [53]), no case–control study revealed a relevant benefit of hydroxychloroquine in pediatric cancer patients. Two studies reported on reconvalescent plasma to treat patients with severe COVID-19 (QoE III_CR_ [22], QoE III_CR_ [54]). However, the Guideline by the Infectious Diseases Working Party limit their recommendation on the effect of reconvalescent plasma (QoE III_CR_) [27, 56]. Systemic steroids, which are an integral component of most leukemia protocols, were reported to be beneficial in three symptomatic SARS-CoV-2-positive patients (QoE III_CR_) [54, 55]. However, without a comparable (at least propensity-matched) control group, this effect may also be difficult to illuminate. Early reports did not often comment on whether treatments directed against COVID-19 had any positive impact on the course of the disease. However, two studies recommended antiviral treatment that could be beneficial in managing severe courses of SARSCoV-2 infection to shorten the time of recovery and allowing earlier administration of chemotherapy (QoE I_U_ [39], QoE III_CR_ [42]).

The decision whether and how to proceed with anticancer treatment remains a major challenge for the attending pediatric oncologists facing patients with a positive SARS-CoV-2 test result. In these patients, the risk of cancer progress or relapse due to interruption of chemotherapy has to be weighed against the risk of severe COVID-19 disease with potentially fatal outcome. The fact that chemotherapy was continued in 14 of 79 patients of our meta-analysis despite SARS-CoV-2 positivity clearly demonstrates the conflict of different aims. Several studies (QoE II_T_ [23], QoE II_U_ [49]) recommend a multidisciplinary decision approach on treatment postponement, modification, or continuation in these situations. To overcome an individualized interdisciplinary clinical and ethical decision process, the characterization of prognostic factors for severe COVID-19 disease courses is recommended in two studies (QoE II_T_ [41], QoE III_CR_ [55, 56]). Unfortunately, blood parameters are only reported sporadically and often lack temporal relation to SARS-CoV-2 infection, which significantly limits further analysis of prognostic factors. In contrast to studies, in which the majority of anticancer treatment was postponed (QoE III_CS_ [15], QoE II_T_ [23], QoE II_T_ [50]), some reports recommend individual chemotherapy modification according to the clinical course of SARS-CoV-2 infection and existing comorbidities (QoE I_U_ [39], QoE II_T_ [41], QoE II_U_ [49]).

In conclusion, our data indicate that SARS-CoV-2 infection in pediatric cancer patients results in a severe clinical course in the minority of patients (QoE II_T_ [41], QoE II_T_ [46]). As most children are infected by a close adult contact (QoE III_CR_ [5], QoE III_CR_ [6], QoE III_CS_ [20], QoE III_CR_ [21], QoE II_U_ [38]), vaccination of adults could be an important strategy (QoE II_T_ [52]). Continuation of chemotherapy in individual pediatric cancer patients with SARS-CoV-2 infection seems possible (QoE II_T_ [23], QoE II_T_ [47]), but more data is needed before solid recommendations can be made. More information on pediatric cancer patients with SARS-CoV-2 infection in prospective national and international data registries would be helpful as well as an international guideline on the management on pediatric patients with SARS-CoV-2 infections.

## Supplementary information

Below is the link to the electronic supplementary material.

Supplementary file1 (XLSX 38 KB)

## Data Availability

The Supplementary material of this review is available in Online Resource 1. All data are available from the corresponding author upon reasonable request.

## Abbreviations

ALL: Acute lymphatic leukemia
AML: Acute myeloblastic leukemia
CNS: Central nervous system
COVID-19: Coronavirus disease 2019
CT: Computer tomography
GVHD: Graft-versus-host disease
HSCT: Hematopoietic stem-cell transplantation
MTX: Methotrexate
PEG asparaginase: Polyethylene glycol-L-asparaginase
SARS-CoV-2: Severe acute respiratory syndrome coronavirus 2
